# Adipose Tissues Characteristics of Normal, Obesity, and Type 2 Diabetes in Uygurs Population

**DOI:** 10.1155/2015/905042

**Published:** 2015-07-27

**Authors:** Jun Zhang, Zhiwei Zhang, Yulei Ding, Peng Xu, Tingting Wang, Wenjing Xu, Huan Lu, Jun Li, Yan Wang, Siyuan Li, Zongzhi Liu, Na An, Li Yang, Jianxin Xie

**Affiliations:** ^1^School of Medicine, Shihezi University, Shihezi, Xinjiang 832000, China; ^2^Department of Endocrinology and Metabolism, First Affiliated Hospital, Shihezi University School of Medicine, Shihezi, Xinjiang 832002, China; ^3^Endocrinology Department of Xinjiang Uygur Autonomous Region People's Hospital, Urumqi, Xinjiang 830001, China; ^4^Department of Endocrinology, Fifth Affiliated Hospital, Xinjiang Medical University, Urumqi, Xinjiang 830011, China

## Abstract

Our results showed that, at the same BMI level, Uygurs have greater WHR values, abdominal visceral fat content, and diabetes risks than Kazaks. In addition, values of HDL-C in Uygur subjects were lower than those in Kazak subjects, and values of creatinine, uric acid, diastolic blood pressure, blood glucose, and fructosamine in Uygur male subjects were lower than those in Kazak male subjects. In contrast, systolic blood pressure values in Uygur subjects were greater than those in Kazak subjects, and blood glucose values were greater in Uygur female subjects than in Kazak female subjects. Additionally, in Uygurs, visceral adipose tissue expression levels of *TBX1* and *TCF21* were greater in obesity group than in normal and T2DM groups and lower in T2DM group than in normal group (*P* < 0.01). The visceral adipose tissue expression levels of* APN* in normal group was greater than those in obesity and T2DM groups, and visceral adipose tissue expression levels of* TNF-α* and* MCP-1* in normal group were lower than those in obesity and T2DM groups (*P* < 0.01). In conclusion, T2DM in Uygurs was mainly associated with not only distribution of adipose tissue in body, but also change in metabolic activity and adipocytokines secretion of adipose tissue.

## 1. Introduction

Diabetes mellitus (DM), also known as simply diabetes, is a chronic and heterogeneous metabolic disorder that affects millions of people worldwide. The previous study showed that the increasing global prevalence of type 2 diabetes mellitus (T2DM) is associated with the rising obesity rates [1], and the obesity is a key factor in the development of T2DM [2]. Yan et al. reported that, for the Uygur and Kazak ethnic groups (i.e., two major ethnic minorities in Xinjiang) at the equal body mass index (BMI) level, more Kazak people developed hypertension, whereas more Uygur people developed diabetes [3]. In addition, the Uygur subjects had significantly greater waist-hip ratio (WHR) than the Kazak subjects, and the Uygur subjects had increased fat distribution in the abdominal viscera, whereas the Kazak subjects had more subcutaneous fat [3]. Additionally, Ibrahim reported that abdominal obesity imparts a greater risk of developing diabetes and future cardiovascular events than peripheral or gluteofemoral obesity, and visceral adipose tissue has a higher rate of insulin-stimulated glucose uptake compared with subcutaneous obesity [4]. Fat depots contribute differently to disease and function [5]. Furthermore, Perrini et al. reported that cytokine release profiles were distinct in the subcutaneous and visceral adipose tissue [6].

Although Uygurs and Kazaks have essentially the same eating habits and living environments, their body fat distributions are different. In the current study, to further explore the correlation of obesity position and T2DM, we examined the characteristic of fat tissues traits and gene expression in visceral adipose tissues of the normal, obesity, and T2DM individuals in Uygurs population.

## 2. Subjects and Methods

### 2.1. Subjects

Our study consisted of 980 Uygur participants (580 males and 400 females) and 1122 Kazak participants (415 males and 707 females) from Yili and Kashi in Xinjiang province of China, and the subjects used in our study are new and different individuals from those in [3]. All patients completed a series of conventional questionnaires, including disease history and daily living and eating habits. In addition, all patients underwent the following measurements: blood biochemical analysis and blood pressure, height, weight, waist, and hip circumference measurements; and for the blood pressure measurement the systolic pressure and diastolic pressure were detected and recorded by measuring 3 tests.

Subsequently, 18 Uygur subjects (9 males and 9 females) and 18 Kazak subjects (9 males and 9 females) aged from 40 to 60 years were randomly selected from the 980 Uygur participants and 1122 Kazak participants, respectively, to have body composition analysis, including determining their overall fat content using the underwater weighing test and MRC measurements of subcutaneous abdominal fat weight and visceral fat. We label-grouped all examined samples into 3 groups (normal, obesity, and diabetes, resp.). Our definition of obesity was determined using the 1999 diagnostic criteria from the World Health Organization and the International Obesity Task Force Asian adult standard from 2000. Specifically, a BMI ≥ 25 kg/m^2^ was defined as obese or overweight, and a BMI < 25 kg/m^2^ was normal. Additionally, all patients with T2DM (2-hour postprandial glucose ≥ 11.1 mmol/L, fasting glucose ≥ 7.0 mmol/L; World Health Organization in 1999) were confirmed by the Xinjiang Uygur Autonomous Region People's Hospital Department.

The intra-abdominal adipose tissues of another 124 Uygur participants, which were contained in the 980 Uygur participants, were collected from Kashi city in Xinjiang province of China: 50 samples for normal control (normal) group, 48 samples for obesity group, and 26 samples for T2DM group. The biopsy of abdominal adipose tissues undergoing surgery was on the protocol to perform pathological analysis, and the mRNA sample taken had been informed before surgery, and every subject accepted to participate by signing a written informed consent.

### 2.2. Methods

#### 2.2.1. Measurement of Biochemical Indexes

One day before elective abdominal operation, the weight, waist, hip, and glycemic index were detected and BMI and WHR were calculated. T2DM was diagnosed according to T2DM diagnostic criteria (2-hour postprandial glucose ≥ 11.1 mmol/L, fasting glucose ≥ 7.0 mmol/L; World Health Organization in 1999). The patients suffering from cancer, acute inflammation, liver and kidney disease, patients with type 1 diabetes, and patients recently taking drugs that may interfere with glucose and lipid metabolism were not included in the present study. The fasting plasma glucose (FPG) was detected using the glucose oxidase method, and total cholesterol (TC), triglyceride (TG), high-density lipoprotein cholesterol (HDL-C), and low density lipoprotein cholesterol (LDL-C) were all detected using automatic biochemistry analyzer.

#### 2.2.2. Body Composition Analysis

Height and weight were measured using standard procedures. Waist and hip circumferences were measured using a flexible tape with tension calipers at the extremity (Gulick-Creative Health Product, Inc., Plymouth, MI), midway between the xiphoid and umbilicus during the midexpiratory phase and at the maximum circumference in the hip area, respectively. Skinfold thickness was measured at 5 different anatomical sites (i.e., subscapular diagonal and vertical, chest, midaxillary, abdominal horizontal and vertical, and suprailiac diagonal and vertical) using Lange Skinfold callipers (Cambridge Scientific Instruments Inc., Cambridge, MD), as previously reported [7]. Truncal skin folds were computed as the sum of the skin folds at these 5 anatomic sites. Body composition was determined using underwater weighing, as previously reported [8]. MRI was used to measure intra-abdominal (visceral) and abdominal subcutaneous adipose tissue volume, as previously described [9]. In brief, MRI studies were performed using a 1.5 T imaging device (Philips Gyroscan Intera, Holland). The entire abdominal region was scanned using contiguous axial 10 mm slices. Fat volume was measured in each slide by mapping subcutaneous and intra-abdominal adipose tissue compartments using computerized images. Volume was converted into adipose tissue mass, assuming an adipose tissue density of 0.9196 kg/L [10].

#### 2.2.3. Tissues Sample

The abdominal omental adipose tissues were collected on the day of surgery, and about 3 cm × 3 cm tissues were collected from each individual, immediately washed with 0.75% NaCl solution, and snap-frozen and stored in liquid nitrogen until RNA extraction.

#### 2.2.4. RNA Isolation and Real-Time PCR

Total RNA was isolated from the visceral adipose tissue (abdominal omental adipose tissue) in the 124 Uygur subjects (50 samples for normal group; 48 samples for obesity group; and 26 samples for T2DM group) by using TRIZOL reagent (Cat#15596-026, Life technologies, Carlsbad, CA, USA) and purified by using an RNeasy minikit (Cat#74106, QIAGEN, GmBH, Germany). RNA integrity was checked on an Agilent 2100 Bioanalyzer (Agilent Technologies, Santa Clara, CA, USA). Reverse transcription conditions for each cDNA amplification were 25°C for 5 min, 42°C for 60 min, and 70°C for 15 min.

Real-time PCR was used to detect gene expression using the SYBR Premix Ex Taq (Takara) on a 7500 Real-Time PCR System (Applied Biosystems, Foster City, CA) with the primers shown in Table 1. Glyceraldehyde 3-phosphate dehydrogenase (GAPDH) was used as internal reference. Part (1 *μ*L) of each RT reaction product was amplified in a 20 *μ*L PCR reaction system. Reaction mixtures were incubated in an ABI Prism 7500 sequence detection system (Applied Biosystems), programmed to conduct 1 cycle at 95°C for 30 s and 40 cycles at 95°C for 5 s and 60°C for 34 s. Moreover, dissociation curves were analyzed using the Dissociation Curve 1.0 software (Applied Biosystems) for each PCR reaction to detect and eliminate possible primer-dimer artifacts. All reactions were performed in triplicate. The relative amounts of target gene transcripts were calculated using the comparative cycle-time method.

#### 2.2.5. Patient Consent and Ethics Statement

All participants were informed of the usage of their basic and clinical information prior to the sample collection, and all participants provided written informed consent for study participation. The consent form and ethical approval were provided by the Medical Ethics Committee at First Affiliated Hospital, Shihezi University School of Medicine (reference number 2014LL22).

#### 2.2.6. Statistical Analysis

The SPSS statistical package (version 11.5, SPSS Inc., Chicago, IL, USA) was used for the data analyses. All data are presented as mean plus standard deviation (mean ± SD). The Shapiro-Wilk test was used to test the normality of data. If the data follow the normality role, differences between groups were analyzed using unpaired Student's *t*-test; else the differences between groups were analyzed using rank sum test. Values of *P* of <0.05 were considered significant unless otherwise specified.

## 3. Results

### 3.1. Clinical Characteristics

The data in Table 2 shows that at similar BMI values, compared with the Kazak subjects, the Uygur subjects had increased values in weight, waist circumference, hip circumference, and WHR. In addition, the values of HDL-C in Uygur male and female subjects were significantly lower than those in Kazak male and female subjects, and the values of creatinine, uric acid, diastolic blood pressure, blood glucose, and fructosamine in Uygur male subjects were significantly lower than those in Kazak male subjects. In contrast, the systolic blood pressure values in Uygur male and female subjects were significantly greater than those in Kazak male and female subjects, and blood glucose values were significantly greater in Uygur female subjects than in Kazak female subjects. Additionally, consistent with the previous report [3], our results also indicated that the Uygurs had a significantly greater risk of diabetes than the Kazaks for both males and females. No significant difference was detected in the values of triglycerides, cholesterol, and LDL-C between both male and female Uygur sbujects and Kazak subjects.

Both 18 individuals from the Uygur and Kazak ethnic subjects (980 and 1122, resp.) and 6 individuals (3 male and 3 female) aged from 40 to 60 in each group (including normal, obesity, and T2DM) were randomly selcected for body composition analysis, and the baseline characteristics for 36 subjects are shown in Table 3. There were no significant differences observed in the ages of the Uygur and Kazak participants. In both Uygur and Kazak ethnic groups, the body weight, BMI, and WHR values of the normal control group were significantly lower than those of the obesity and T2DM groups (*P* < 0.05). Notably, although there was no significant difference in total lipid content between subjects from the two ethnic groups, the ratio of VF/SAF in Uygurs was significantly higher than that of the Kazaks in both obesity and T2DM groups (*P* < 0.05). However, there was no difference between the two ethnic groups regarding fasting blood glucose levels.

### 3.2. Candidate Gene Expression in the Visceral Adipose Tissue of Normal, Obesity, and T2DM Individuals in Uygurs Population

**Figure 1:**
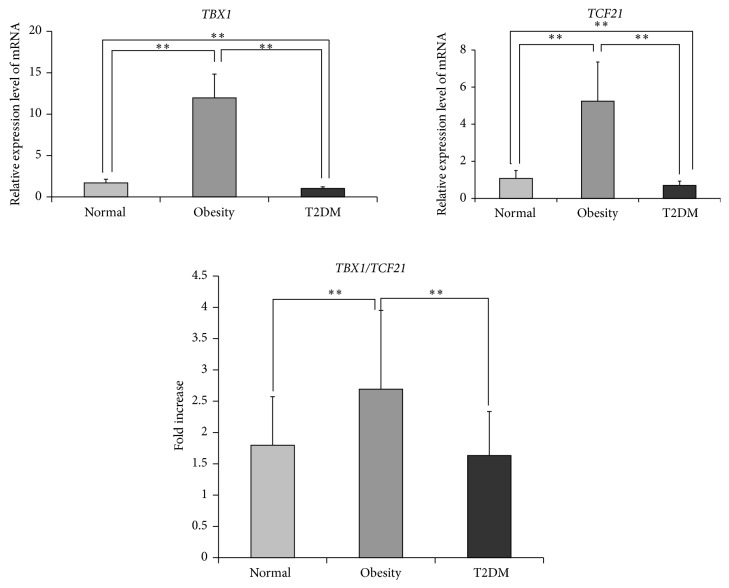
The mRNA expression levels of T-box protein 1 (*TBX1*) and transcription factor 21 (*TCF21*) in the visceral adipose tissue of Uygur population. Comparison between groups by *t*-test, ^**^ *P* < 0.01; *n* = all subjects in each group (50, 48, and 26 for the normal, obesity, and T2DM groups, resp.); T2DM, type 2 diabetes mellitus.

To reveal the function change of the adipose tissues among normal, obesity, and T2DM individuals, five candidate genes' expressions in visceral adipose tissue of normal, obesity, and T2DM individual in Uygur population were analyzed using real-time PCR. The result showed that five candidate genes, including T-box protein 1 (*TBX1*), transcription factor 21 (*TCF21*), adiponectin (*APN*), tumor necrosis factor-alpha (*TNF-α*), and monocyte chemotactic protein 1 (*MCP-1*), were all expressed in the visceral adipose tissue of normal, obesity, and T2DM individuals in Uygur population. The* TBX1* and* TCF21* expression levels in the obesity group were significantly higher than those of normal and T2DM groups (*P* < 0.01); and the* TBX1* and* TCF21* expression levels in the T2DM group were significantly lower than those of normal and obesity groups (Figure 1, *P* < 0.01). In addition, statistical analysis showed that the ratio of expression level of* TBX1* to* TCF21* (*TBX1*/*TCF21*) in obesity group was significantly greater than those in normal and T2DM groups (Figure 1, *P* < 0.01). Additionally, the expression of* APN* in the normal individual was significantly higher than those in obesity and T2DM groups, the expression levels of* TNF-α* and* MCP-1* in normal group were significantly lower than those in obesity and T2DM groups (Table 4, *P* < 0.05), and no significant differences of* APN*,* TNF-α*, or* MCP-1* expression were detected between obesity and T2DM groups (Table 4, *P* > 0.05).

## 4. Discussion

The previous report showed that visceral and subcutaneous adipocytes may exhibit different properties in the production of bioactive molecules [11]. In the current study, the results showed that, at the same BMI level, both male and female Uygur subjects have significantly greater values in WHR, abdominal visceral fat content, and diabetes risks, compared with Kazak subjects. This result is consitent with the previouse study [3].

In addition, the values of HDL-C in Uygur male and female subjects were significantly lower than those in Kazak male and female subjects, and the values of creatinine, uric acid, diastolic blood pressure, blood glucose, and fructosamine in Uygur male subjects were significantly lower than those in Kazak male subjects. In contrast, the systolic blood pressure values in Uygur male and female subjects were significantly greater than those in Kazak male and female subjects, and blood glucose values were significantly greater in Uygur female subjects than in Kazak female subjects. These results showed that the metabolic difference result from obesity exists between the Uygurs and Kazaks populations.

Toyoda et al. [12] reported that visceral adipose tissue is a better predictor for mortality than subcutaneous tissue. Our results showed that gene expression in the visceral adipose tissue of normal, obesity, and T2DM subjects in the Uygurs was significantly different, and the expression levels of* TBX1* and* TCF21*, the marker of white adipocytes and brown-like white adipocytes, respectively [13], were significantly greater in the obesity group than in normal and T2DM groups and significantly lower in the T2DM group than in normal group (*P* < 0.01). These results suggested that the lipid metabolic activity of visceral adipose among normal, obesity, and T2DM subjects was different in the Uygurs population. The ratio of expression level of* TBX1* to* TCF21* (*TBX1*/*TCF21*) in obesity group was significantly greater than those in normal and T2DM groups (*P* < 0.01), suggesting that the lipid metabolic activity of visceral adipose tissue in obesity individuals was lower than those in normal and T2DM individuals.

APN is an adipocytokine produced by adipose tissue and plays a protective role in vascular injury and insulin resistance (IR). The previous study in other ethnic groups showed that the subjects with diabetes mellitus (DM) had greater insulin and lower APN level [14], and Asian participants, who suffered from higher chronic disease risk for obesity, had lower serum levels of APN than Caucasian participants across all levels of BMI [15]. Our result showed that the visceral adipose tissue expression levels of* APN* were lower in obesity and T2DM groups than in normal group; this is consistent with the previous study [14] and suggested that downregulation of* APN* might be a reason for the obesity and T2DM.

TNF-*α* and MCP-1 are two proinflammatory cytokines that can be released by adipose tissue. In the current study, the results showed that the visceral adipose tissue expression levels of* TNF-α* and* MCP-1* in normal group were significantly lower than those in obesity and T2DM groups (*P* < 0.01), which were consistent with the previous study where TNF-*α* and MCP-1 were elevated in poor glycemic control and good glycemic control overweight and obese patients [16], suggesting that the immune response of visceral adipose tissue was changed during the occurrence of obesity and T2DM.

Collectively, our result indicated that the T2DM in the Uygurs population was mainly associated with not only distribution of adipose tissue in the body, but also the change in metabolic activity and adipocytokines secretion of adipose tissue.

## Figures and Tables

**Table 1:** Primers used in RT-qPCR.

Gene	Sequence ID	Primer (5′-3′)	Product (bp)
*APN *	NM_004797.3	F: ATGGCCCCTGCACTACTCTA	104
		R: CAGGGATGAGTTCGGCACTT	

*TNF-α*	NM_000594.3	F: GTGACAAGCCTGTAGCCCAT	111
		R: TATCTCTCAGCTCCACGCCA	

*MCP-1 *	NM_002982.3	F: GATCTCAGTGCAGAGGCTCG	105
		R: TTTGCTTGTCCAGGTGGTCC	

*TCF21 *	NM_003206.3	F: GCAGATCCTGGCTAACGACA	134
		R: TGGTTCCACATAAGCGGCTC	

*TBX1 *	XM_005261271.1	F: AACCTACTGGACGACAACGG	189
		R: CTGCGTGATCCGATGGTTCT	

*GAPDH *	NM_001256799.1	F: TGTTGCCATCAATGACCCCTT	202
		R: CTCCACGACGTACTCAGCG	

**Table 2:** Basic indices and biochemical analyses.

	Uygur		Kazak	
	Males (580)	Females (400)	Males (415)	Females (707)
Age (years)	44.09 ± 15.99	39.5 ± 14.05	41.25 ± 15.06^**^	38.31 ± 14.2
Diabetes prevalence rate (%)	6.7	7.9	1.2^**^	0.7^**^
Height (cm)	168.46 ± 7.3	157.09 ± 6.15	170.54 ± 7.53^**^	159.49 ± 6.43
Weight (kg)	74.19 ± 14.23	63 ± 11.79	69.13 ± 12.64^**^	59.78 ± 10.75^**^
BMI (kg/m^2^)	26.22 ± 5.24	25.55 ± 4.65	23.77 ± 4.13	23.52 ± 4.15
Waist circumference (cm)	93.84 ± 12.01	90.06 ± 18.45	89.52 ± 12.36^**^	83.91 ± 14.27^**^
Hip circumference (cm)	101.55 ± 8.77	101.23 ± 15.02	100 ± 9.01^*^	97.3 ± 13.07^*^
WHR	0.92 ± 0.08	0.89 ± 0.09	0.89 ± 0.07^**^	0.86 ± 0.08^*^
Systolic blood pressure (mmHg)	129.74 ± 20.95	125.16 ± 24.32	128.98 ± 23.97^**^	120.52 ± 22.61^**^
Diastolic blood pressure (mmHg)	82.82 ± 14.17	80.88 ± 15.63	83.6 ± 14.29^**^	80.28 ± 15.20
Creatinine	59.45 ± 7.81	57.17 ± 6.13	64.10 ± 12.75^**^	53.2 ± 9.26
Uric acid	255.0 ± 44.55	230.08 ± 65.10	271.83 ± 39.89^**^	221.03 ± 53.62
Glucose (mmol/L)	4.83 ± 0.73	5.14 ± 0.72	4.91 ± 0.51^**^	4.74 ± 0.63^*^
Fructosamine	2.32 ± 0.08	2.37 ± 0.15	2.39 ± 0.16^*^	2.38 ± 0.2
Triglycerides (mmol/L)	1.85 ± 1.52	1.51 ± 1.41	1.09 ± 0.56	1.05 ± 0.56
Cholesterol (mmol/L)	4.13 ± 1.62	3.80 ± 1.92	4.38 ± 1.37	4.58 ± 1.32
LDL-C (mmol/L)	2.13 ± 1.04	2.18 ± 1.02	2.54 ± 1.08	2.56 ± 1.01
HDL-C (mmol/L)	1.54 ± 0.33	1.54 ± 0.36	1.61 ± 0.36^*^	1.84 ± 0.46^**^

BMI as covariance, all indices were executed as *t*-tests between two ethnic subjects both male and female. HDL: high density lipoproteins; LDL: low density lipoproteins. Values are given as the mean ± SD. ^**^ *P* < 0.01; ^*^ *P* < 0.05.

**Table 3:** Baseline characteristics of the subjects.

	Uygur			Kazak		
	Normal (6^#^)	Obesity (6)	T2DM (6)	Normal (6)	Obesity (6)	T2DM (6)
Age (y)	41 ± 6	40 ± 2	50 ± 6	46 ± 6	39 ± 5	42 ± 6
Body weight (kg)	59.0 ± 6.33^*^	75.10 ± 14.01	80.17 ± 6.56	63.40 ± 10.32^*^	79.67 ± 13.11	82.43 ± 15.23
BMI (kg/m^2^)	23.46 ± 1.55^*^	38.06 ± 1.922	36.27 ± 2.97	23.77 ± 3.52^*^	31.74 ± 1.73	33.43 ± 1.83
WHR	0.886 ± 0.490^*^	0.948 ± 0.325	0.953 ± 0.451	0.867 ± 0.470^*^	0.951 ± 0.324	0.944 ± 0.534
Total lipid contents (%)	0.214 ± 0.123	0.301 ± 0.118	0.292 ± 0.145	0.226 ± 0.098	0.318 ± 0.157	0.298 ± 0.088
VF/SAF	0.46 ± 0.22	0.56 ± 0.18^&^	0.54 ± 0.13^&^	0.42 ± 0.19	0.40 ± 0.22	0.39 ± 0.10
Fasting blood glucose (mg/dL)	4.58 ± 0.24	5.67 ± 0.15	9.89 ± 2.44	4.29 ± 0.18	6.24 ± 0.48	9.44 ± 2.86

T2DM, type 2 diabetes mellitus; VF, visceral fat; SAF, subcutaneous abdominal fat; all such values were expressed as mean ± SD (*n* = 6; 3 males and 3 females, resp.); ^#^the number of subjects in one group; ^*^ *P* < 0.05 in the same ethnic group; ^&^ *P* < 0.05 in different ethnic group.

**Table 4:** Comparison of adipose cytokines mRNA copy data in the visceral adipose tissue of Uygur population (rank sum test).

	*APN *	*TNF-α*	*MCP-1 *
Normal (50^#^)	0.7162^*^ (0.5668–0.9564)	0.0250^*^ (0.0195–0.0672**)**	0.1588^*^ (0.0872–0.2663)
Obesity (48)	0.4244 (0.2209–0.6004)	0.1096 (0.0637–0.1592)	0.1937 (0.0915–0.3346)
T2DM (26)	0.4120 (0.1967–0.5560)	0.0798 (0.0569–0.1428)	0.1983 (0.1315–0.4083)

^*^ *P* < 0.05; ^#^the number of subjects in one group.
